# A validation study of the ICECAP-O in informal carers of people with dementia from eight European Countries

**DOI:** 10.1007/s11136-019-02317-3

**Published:** 2019-10-08

**Authors:** Meg Perry-Duxbury, Job van Exel, Werner Brouwer, Anders Sköldunger, Manuel Gonçalves-Pereira, Kate Irving, Gabriele Meyer, Geir Selbæk, Bob Woods, Orazio Zanetti, Frans Verhey, Anders Wimo, Ron L.H. Handels, The Actifcare Consortium

**Affiliations:** 1 Erasmus School of Health Policy & Management, Erasmus University Rotterdam, Rotterdam, The Netherlands; 2 Erasmus School of Economics, Erasmus University Rotterdam, Rotterdam, The Netherlands; 3 Karolinska Institutet, Department for Neurobiology, Care Sciences and Society, Division of Neurogeriatrics, Solna, Sweden; 4 CEDOC, Chronic Diseases Research Centre NOVA Medical School/Faculdade de Ciências Médicas, Universidade Nova de Lisboa, Lisbon, Portugal; 5 School of Nursing and Human Sciences, Dublin City University, Dublin, Ireland; 6 Medical Faculty, Institute for Health and Nursing Science, Martin Luther University Halle-Wittenberg, Magdeburger Straße 8, 06112 Halle (Saale), Germany; 7 Norwegian National Advisory Unit ON Ageing and Health, Vestfold Health Trust, Tønsberg, Norway; 8 Department of Geriatric Medicine, Oslo University Hospital, Oslo, Norway; 9 Faculty of Medicine, Institute of Clinical Medicine, University of Oslo, Oslo, Norway; 10 Dementia Services Development Centre Wales (DSDC), Bangor University, Bangor, UK; 11 Alzheimer Center Limburg, Faculty of Health, Medicine and Life Sciences, School for Mental Health and Neuroscience, Department of Psychiatry and Neuropsychology, Maastricht University Medical Center +, Maastricht, The Netherlands; 12 Centre for Research & Development, Uppsala University/County Council of Gävleborg, Gävle, Sweden; 13 IRCCS Istituto Centro San Giovanni di Dio Fatebenefratelli, Brescia, Italy

**Keywords:** Construct validity, ICECAP-O, Informal care, Dementia, Well-being

## Abstract

**Purpose:**

The pressure on healthcare budgets remains high, partially due to the ageing population. Economic evaluation can be a helpful tool to inform resource allocation in publicly financed systems. Such evaluations frequently use health-related outcome measures. However, in areas such as care of older people, improving health outcomes is not necessarily the main focus of care interventions and broader outcome measures, including outcomes for those providing informal care, may be preferred when evaluating such interventions. This paper validates a recently introduced well-being measure, the ICECAP-O, in a population of informal carers for people with dementia from eight European countries.

**Methods:**

Convergent and discriminant validity tests were performed to validate the ICECAP-O using data obtained in a sample of 451 respondents from Germany, Ireland, Italy, the Netherlands, Norway, Portugal, Sweden and the UK. These respondents completed a number of standardized questionnaires within the framework of the Actifcare project.

**Results:**

The ICECAP-O performed well among informal carers, in terms of both convergent and discriminant validity. In the multivariate analysis, it was found to be significantly associated with the age of the person with dementia, EQ-5D-5L health problem index of the person with dementia, carer–patient relationship, care recipient CDR, carer LSNS Score, the PAI score, and Perseverance Time.

**Conclusion:**

The ICECAP-O appears to be a valid measure of well-being in informal carers for people with dementia. The ICECAP-O may therefore be useful as an outcome measure in economic evaluations of interventions aimed at such informal carers, when these aim to improve well-being beyond health.

## Introduction

Dementia is highly prevalent in today’s society. It was estimated that worldwide 50 million people lived with dementia in 2018, and with the ageing population this number is expected to increase to 82 million by 2030 and further to 152 million by 2050 [1]. With these increasing numbers comes an increase in the magnitude of care required. Family members, who are most often elderly spouses, siblings or friends, are frequently the ones to provide (part of) this care [2]. These informal carers may be unprepared for the physical and emotional demands that caring entails and many carers experience considerable strain and well-being losses due to their caregiving tasks [3, 4].

At the same time, governments struggle with the rising demand for care of people with dementia, those with other (chronic) diseases, and their carers, and with limited health care resources. Regarding decisions for optimal spending, economic evaluation is a useful decision-making aid. It is increasingly applied in many Western healthcare systems. Usually in economic evaluations, health-related quality of life (HrQoL) is used to measure intervention benefits. However, in areas such as mental health and care of older people, improving health outcomes is not necessarily the main focus of care interventions [5, 6]. Broader outcome measures, which go ‘beyond health’ [7], capturing the effects of interventions in terms of well-being, for both patients and carers, may be more appropriate to capture relevant benefits of such interventions.

Several well-being measures have recently been developed, broadening the evaluative scope beyond health in different ways. Some of these have focused on context-specific elements of well-being beyond health, such as care-related quality of life in carers [8, 9] or social care-use-related well-being [10]. Analogous with measuring disease-specific quality of life rather than generic health-related quality of life, context-specific measures may be more sensitive to specific changes, but this comes at the expense of comparability across situations and populations. Generic measures of well-being in principle allow comparisons, across interventions, diseases and situations. The ICECAP-O, a capability well-being measure for older people, is such a generic well-being measure [11], which is increasingly used. Capability in this context refers to the extent to which a person is able to function in a particular way, whether or not he or she chooses to do so [11]. The ICECAP-O consists of five important general capability well-being dimensions: attachment, security, role, enjoyment and control, and has four answering levels per domain. Values for the states described with the instrument were derived from a sample of older people in England, using best–worst scaling [12]. ICECAP-O questionnaires and further information can be found on the website: www.birmingham.ac.uk/research/activity/mds/projects/HaPS/HE/ICECAP/ICECAP-O.

As a generic well-being measure, the ICECAP-O is well suited for economic evaluation of care interventions in elderly populations, especially those who are suffering from chronic diseases [13]. Given the generic nature of the instrument, it may be suitable to measure well-being not only in patients but also in informal carers. Using the ICECAP-O to measure well-being in both patients and their informal carers would facilitate comparisons and aggregation of outcomes within the same economic evaluation. This is also relevant for a scenario in which two interventions are being compared, and one of these interventions impacts informal carers. Without a comparable outcome between patients and informal carers, for example if the ICECAP-O and the CarerQoL were used, respectively, it would be necessary to perform multiple-criteria decision analysis, which may be more costly and time consuming.

Before the ICECAP-O can be used as a well-being measure in economic evaluations of care interventions, it needs to be validated in relevant populations. Validation is performed to assess the extent to which a measure evaluates what it sets out to represent. The most frequently used validity tests for health status measures in the literature are construct validity tests. These examine the extent to which the measure indeed captures the concept it intends to measure [14]. Construct validity consists of both convergent and discriminant validity. Convergent validity refers to the extent to which a measure correlates with related concepts [14], while discriminant validity refers to the extent to which relevant differences in (sub-) groups are adequately reflected by the measure [9].

So far, the ICECAP-O has been validated for various populations such as older people in England [12, 15–18], psycho-geriatric older people in nursing homes in the Netherlands [19], post-hospitalized older people in the Netherlands [20] and older people with dementia in Germany [5], mostly with favourable results. In these studies, sample sizes typically have been relatively small and taken from only one country. To date, to our knowledge, no study has validated the ICECAP-O as a well-being measure in informal carers.

In this study, we therefore add to the literature in a number of ways. This is the first study to validate the ICECAP-O in a sample of informal carers, using a rich dataset. We use data from a relatively large sample of carers obtained in eight European countries in the context of the Actifcare project [21], which aims to analyse the pathways to care for people with dementia and their families. This paper therefore considers the construct validity of the ICECAP-O in a sizeable international population of informal carers for people with dementia. Furthermore, validating the ICECAP-O in this kind of population allows those performing economic evaluations to consider the ICECAP-O when measuring carer well-being.

## Methods

Data were collected in eight European countries: Germany, Ireland, Italy, the Netherlands, Norway, Portugal, Sweden and the UK. Care receivers adhering to the specified criteria ("Appendix 1") and their informal carers were invited to complete the questionnaires, available in seven different languages. For all measures, including the ICECAP-O, nationally validated versions were used, or, if not available, the measure was translated, back translated and pilot tested following a translation protocol [21–23]. The data collection consisted of different parts. People with dementia and carers were interviewed by trained interviewers about their socio-demographic characteristics and the comorbidities and health care resource use of the former. Carers completed questionnaires covering a variety of outcome measures and were interviewed about the caregiving situation, their resource use, and the person with dementia’s health. Finally, the interviewer completed questionnaires about the health, quality of life and care needs of the person with dementia [21].

Demographic characteristics included age, gender, nationality, ethnicity, marital status, level of education and state of employment. Before listing the health and well-being measures used in our analyses, it is worth describing the ICECAP-O in further detail. As mentioned above, the ICECAP-O is a general capability well-being measure, consisting of five dimensions: attachment, security, role, enjoyment and control. These dimensions are sometimes described in a little more detail as ‘love and friendship’, ‘thinking about the future’, ‘doing things that make you feel valued’, ‘enjoyment and pleasure’ and ‘independence’. The ICECAP-O and has four answering levels per domain which are no capability, a little capability, a lot of capability and full capability. The ICECAP-O provides us with separate scores for each domain, meaning there are 1024 different possible ‘capability states’. By attaching the designated utilities to each attribute, we are provided with a final ICECAP-O tariff score with a range between zero (no capability) and one (full capability) [15].

There are health and well-being measures used in our analysis to test the validity of the ICECAP-O. The first measures are those answered by the carer, about the carer and their environment. These are CarerQol [9], EQ-5D-5L [24], Positive Affect Index (PAI) [25], Perseverance Time (PT) [26] and the Lubben Social Network Scale (LSNS) [27]. The next measures are those answered by the carer and/or an interviewer as a proxy about the person with dementia. These are Clinical Dementia Rating (CDR) [28], DemQoL-U [29, 30] (proxy-rated), Quality of Life in Alzheimer’s disease (QoL-AD) [31], Resource Utilization in Dementia (RUD) [32], and finally a subset of questions regarding unmet need from the Camberwell Assessment of Need for the Elderly (CANE) [33]. These measures are discussed and referenced in Table 1. Here it is important to note that when referring to ‘CANE Unmet Need’ we are referring to a measure taken from the CANE instrument, which in this case was collected by an interviewer talking with the carer and person with dementia. In the measure, we sum the number of times ‘unmet need’ is chosen out of the 24 questions asked. Summary statistics of all continuous variables used are shown in "Appendix 2".

**Table 1:** Measures

Measure	Aim	Dimensions/information used	Range
EQ-5D-5L	To measure health-related quality of life [24]	Mobility, self-care, usual activities, pain/discomfort, anxiety/depression. [24] Each dimension has five response categories ranging from ‘no problems’ to ‘severe problems’ [43]	Can be converted to a single utility score with range − 0.281 to 1, with 0 equal to being dead and 1 equal to perfect health. Can also be converted to a single health problems index by summing the scores on the five levels, with range of 0 to 20, with 0 being no health problems [44]
EQ-VAS	To measure health-related quality of life.	One visual analogue scale which patients use to place their ‘self-rated health’ [45]	Range of 0 to 100 where endpoints are labelled ‘worst imaginable health state’ (0) and ‘best imaginable health state’ (100) [45]
CarerQol-7D	To measure and value the impact of providing informal care on carers [9]	Relational problems, mental health problems, problems combining daily activities with care, financial problems, physical health problems, fulfillment from caregiving, support with lending care [9]	Weighted sum score with range 0 to 100, where 100 is the best caregiving situation [46]
CarerQol-VAS	To measure the self-reported happiness of carers [9]	One visual analogue scale which respondents use to rate their ‘self-rated happiness’ [9]	Range of 0 to 10 where 0 is ‘completely unhappy’ and 10 is ‘completely happy’ [9]
PAI	To measure relationship quality [25]	Five items that reflect the respondent’s feelings towards their relative/friend: communication, understanding, trust, fairness, respect and affection [25]	A range of 6 to 30, with 30 being the best score [25]
CDR	To stage the progression of dementia [27]	Uses information provided by patient and carer [27]	Can be one of five stages: (0) no dementia, (0.5) very mild, (1) mild, (2), severe, (3) severe [47]
DemQoL-U	To evaluate health-related quality of life in mild to moderate dementia [28, 29]	Information taken from questionnaire responses [28, 29]	A range of 0 to 1, with 1 being full health [28, 29]
QoL-AD	To describe the relationship of QoL to demographic characteristics, cognitive and functional status, depression and pleasant level activity [30]	13-item self- and proxy-report [30]	A range of 13 to 52, with 52 being the best outcome [30]
CANE Unmet Need	To identify needs for old age people with a mental illness or cognitive problems [33]	24 domains (bio-psycho-social needs), and 2 items referring to carer needs [33]. Original with responses being (0) no need, (1) met need, (2) unmet need	We sum the number of times ‘unmet need’ is chosen out of the 24 questions asked, thereby giving a range of 0 to 24
LSNS	To gauge social isolation in older adults [26]	Measures perceived social support received by family and friends [26]	A range of 0 to 30, where a score of 12 or less suggests being at risk of social isolation [26]
RUD	To assess formal and informal resource use [31]	Number of carers involved in a patient’s care, carer time, carer work status, and patient and carer health resource utilization [31]	N/A
PT	To measure the period of time at which the informal carer indicates to be able to maintain current care if the situation remains the same [32]	Six levels: (1) 1 week or less, (2) between 1 week and 1 month, (3) between 1 and 6 months, (4) between 6 months and 1 year, (5) between 1 and 2 years, and (6) more than 2 years [32]	A range of ‘1 week or less’ to ‘more than 2 years’

### Data analysis

To test whether the ICECAP-O is a valid measure of capability well-being, two main sections of analysis were performed: convergent validity and discriminant validity. A priori expected correlations and relationships between the ICECAP-O and other variables from the questionnaires, discussed below, were drawn from previous literature, if available. In the analyses, correlation strength levels were taken from Cohen’s Set Correlation and Contingency Tables [34]. Correlations are considered strong if the coefficient is above 0.5, moderate if the coefficient is between 0.3 and 0.5, and weak if the coefficient is below 0.3. A *p* value of 0.05 was taken to signify statistical significance.

### Convergent validity

To test convergent validity, Spearman correlation coefficients of the tariff scores and dimensions of the ICECAP-O were compared against the EQ-5D-5L results (utility tariff, health problems index, and VAS) [35], CarerQol-7D tariff scores and CarerQol-VAS scores, respectively. It was anticipated that there would be a moderate positive correlation between the ICECAP-O scores and the EQ-5D-5L utility tariff scores and VAS scores of carers, a moderate negative correlation between the ICECAP-O scores and the EQ-5D-5L health problems index of carers, and a strong positive correlation between the ICECAP-O and the CarerQol scores.

### Discriminant validity

For discriminant validity, sub-groups were defined based on characteristics that previously were shown to be related to informal carer outcomes. For measures that have no pre-defined cut-off points for high or low, in this case the EQ-5D-5L tariff and VAS scores, the cut-off points between sub-groups were primarily based on a face valid classification in relatively similar group sizes. Education was split unto three sub-groups based on primary school only (low), up to high-school education (medium), and higher education (high).

Student’s* t* tests (for two sub-groups) or ANOVA (for more than two sub-groups) were performed to identify significant differences in ICECAP-O scores. Then, a multivariate regression model was estimated for the ICECAP-O tariff scores using all variables in which the ICECAP-O could discriminate at a *P* value of 0.1 or less, to gain insight into the magnitude and significance of the variables that were associated with the ICECAP-O scores. There are exceptions to this exclusion rule: the variables age, gender, education, relationship between the carer and person with dementia, and carer daily hours. We include age, gender, education, and the type of relationship because these are basic demographic factors. It was pre-defined by the authors that carer daily hours would be included in the multivariate regression as it is a key variable in the care giving context. A second model was estimated including country dummies, to account for country-level effects. In this regression, Germany was used as the reference country as it had the lowest mean ICECAP-O score among carers.

Several hypotheses were generated regarding carer, care receiver and caregiving context variables and their relationship with the ICECAP-O. It is important to note that this literature did not necessarily refer to informal caregivers, or carers of people with dementia. Regarding carer variables, employed carers were expected to have a significantly higher ICECAP-O score than those unemployed [36], carers with higher health status (i.e. a higher EQ-5D-5L score) were expected to have significantly higher ICECAP-O scores than those with lower health status [37], and carers with a higher PAI score were expected to have a significantly higher ICECAP-O score than those with a lower PAI score [38]. Furthermore, there was insufficient evidence to form a hypothesis on the effect of carer age on the ICECAP-O [20, 39]. There was no expectation for the ICECAP-O to score differently for different levels of carer education [20]. Regarding care receiver variables, carers for a person with dementia with a higher health status (i.e. a higher EQ-5D-5L, DemQoL-U and QoL-AD score, or a lower CDR score) were expected to have a significantly higher ICECAP-O score than carers for persons with lower health status. Finally, regarding caregiving context variables, carers with a low care burden (i.e. fewer daily care hours, lower CANE unmet needs in the person with dementia, higher PT and/or higher RUD scores) were expected to have a significantly higher ICECAP-O score than those with a higher care burden [40], and carers with a higher LSNS score were expected to have a significantly higher ICECAP-O score than those with a lower LSNS score [41].

All tariff scores (for ICECAP-O, EQ-5D-5L and CarerQol-7D) were calculated using UK value sets because of both their availability and the need for consistency. The proxy ratings of the informal carers were used for the EQ-5D-5L, QoL-AD and DemQoL-U of people with dementia. All analyses were performed in STATA 14.

## Results

### Study sample

A total of 451 informal carers and home-dwelling people with mild to moderate dementia completed the questionnaires and were included in the analysis. The people with dementia were selected for this study based on their probability of needing formal care within 1 year. Table 2 presents sample characteristics of informal carers, the people with dementia (or care receivers) and the caregiving situation. The mean age of informal carers was 66.4 years old. Most of the carers were female and 28% of the carers were employed. The mean age of the care receivers was 77.7 years and approximately half of them were female.

**Table 2:** Sample characteristics and bivariate results

Demographic	%	Mean ICECAP-O tariff	*P* value
**Carer**			
Age			
< 66 years	42.3	0.80	0.01*
≥ 66 years	57.7	0.77	
Gender			
Male	33.6	0.79	0.48
Female	66.4	0.78	
Years of education			
Low (< 8)	19.6	0.73	0.37
Medium (≥ 8 & ≤ 16)	60.8	0.79	
High (> 16)	19.6	0.80	
Occupation			
Employed	28.1	0.84	0.02*
Not employed	71.9	0.76	
Positive affect index			
Low (≤ 21)	51.3	0.75	< 0.01*
High (> 21)	47.7	0.82	
CANE unmet need (0 to 24)			
Low care need (< 2)	55.7	0.79	< 0.01*
Medium care need (≥ 2 and ≤ 5)	34.6	0.80	
High care need (> 5)	9.7	0.69	
Perseverance time			
< 2 years	29.7	0.72	< 0.01*
≥ 2 years	70.3	0.81	
LSNS			
Danger social isolation	24.5	0.71	< 0.01*
No danger social isolation	75.5	0.81	
**Care receiver**			
Age			
< 80 years	51.9	0.78	0.08*
≥ 80 years	48.1	0.79	
Gender			
Male	45.5	0.75	0.62
Female	54.5	0.81	
EQ-5D-5L utility tariff score			
Low (< 0.68)	32.8	0.73	0.01*
Medium (≥ 0.68 and < 0.8)	33.7	0.80	
High (≥ 0.8)	33.5	0.81	
EQ-5D-5L health problems index			
Low (< 6)	62.8	0.81	0.04*
Medium (≥ 6 and ≤ 12)	32.0	0.74	
High (> 12)	0.05	0.50	
EQ-VAS			
Low (< 50)	38.4	0.77	< 0.11
Medium (≥ 50 and ≤ 75)	36.2	0.78	
High (> 75)	25.4	0.80	
QoL-AD			
Low (≤ 31.5)	47.6	0.73	< 0.01*
High (> 31.5)	52.4	0.82	
DemQoL-U			
< 0.9	74.9	0.77	0.08*
≥ 0.9	25.1	0.82	
CDR			
≤ 1	80.4	0.79	0.01*
> 1	19.6	0.74	
RUD (per month)			
Some hospital days	3.0	0.74	< 0.01*
No hospital days	97.0	0.78	
Some practitioner visits	72.1	0.79	0.62
No practitioner visits	27.9	0.78	
Some home care services	72.0	0.79	0.08*
No home care services	27.0	0.78	
**Caregiving situation**			
Relationship with person with dementia			
Spouse/partner	63.9	0.76	0.22
Son/daughter (in-law)	31.9	0.82	
Other	4.2	0.86	
Daily care hours			
Low (< 4 h)	48.5	0.80	0.28
High (≥ 4 h)	51.5	0.77	

*P value of 0.1 or less

**Fig. 1:**
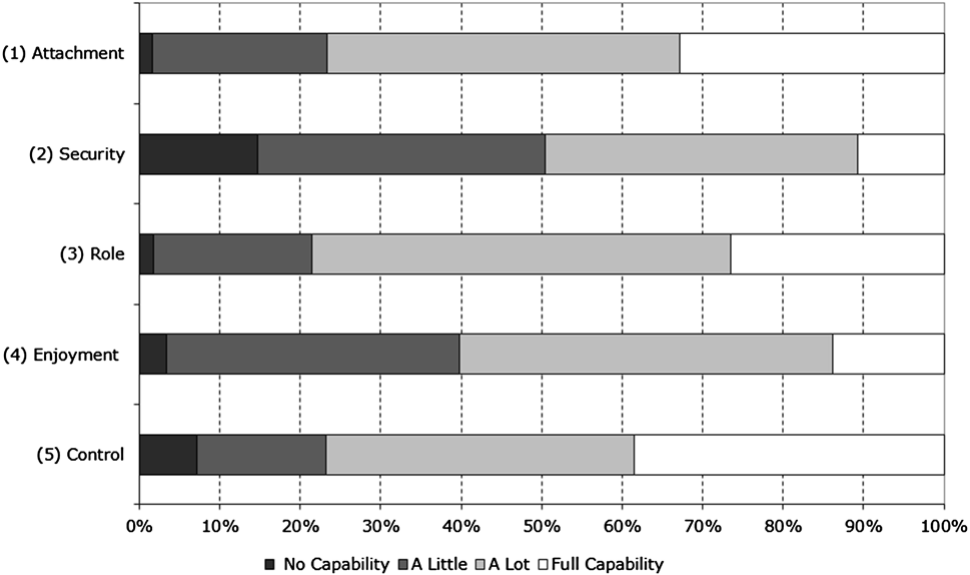
ICECAP-O response of informal carers

Figure 1 shows the scores of informal carers on the different dimensions of the ICECAP-O. The mean ICECAP-O tariff score of the informal carers was 0.78, with standard deviation 0.16. The minimum tariff score in the sample was 0 while the maximum score was 1. The mean ICECAP tariff scores varied per country, as displayed in Table 3.

**Table 3:** ICECAP-O values per country, ranked by mean tariff*

Country	ICECAP-O tariff
Germany	0.732
Portugal	0.753
United Kingdom	0.769
Italy	0.772
Ireland	0.781
The Netherlands	0.809
Sweden	0.816
Norway	0.834

*Differences are statistically significant from each other. *P* value < 0.01

### Convergent validity

The Spearman’s correlation coefficients are presented in Table 4. There was a moderate positive correlation between the ICECAP-O tariff scores and the EQ-5D-5L utility tariff and EQ-VAS scores, a moderate negative correlation with the EQ-5D-5L health problems index, and a strong positive correlation with the CarerQol tariff and CarerQol-VAS scores.

**Table 4:** Spearman correlations

Instrument	ICECAP-O tariff	*P* value	ICECAP-O domains				
			Attachment	Security	Role	Enjoyment	Control
EQ-5D-5L utility tariff score	0.46	< 0.01	0.22	0.42	0.39	0.41	0.26
EQ-5D-5L health problems index	− 0.45	< 0.01	− 0.21	− 0.41	− 0.38	− 0.39	− 0.28
EQ-VAS	0.45	< 0.01	0.25	0.34	0.33	0.42	0.19
CarerQol tariff	0.53	< 0.01	0.38	0.42	0.44	0.46	0.27
CarerQol-VAS	0.54	< 0.01	0.43	0.41	0.46	0.51	0.18

Looking at the dimensions of the ICECAP-O in Table 4, it is clear that the other measures hold the strongest correlations with the Security, Role and Enjoyment dimensions of the ICECAP-O. Country-specific correlations are provided in "Appendix 3". Overall country-specific correlations matched those of the aggregate results, with Sweden being somewhat of an exception. In the correlation results for Sweden, the EQ-5D-5L utility tariff score and health problems index were uncorrelated with the ICECAP-O.

### Discriminant validity

Bivariate results regarding discriminant validity are shown in Table 2. The ICECAP-O significantly discriminated between old and young informal carers, between those who were employed and unemployed, between carers with low and high PAI, between carers who were and were not in danger of social isolation (LSNS) and carers who felt they could and could not continue caregiving for 2 years or more (PT). The ICECAP-O mean scores all differed in the expected direction. The ICECAP-O did not discriminate between carers who had daily care hours of less than 4 h or 4 h and over.

The ICECAP-O discriminated between carers of people who were 80 years of age or over, or below 80 years of age, between carers of those who received some home care services versus those who received no home care services, carers of people with dementia with high and low numbers of unmet needs (CANE), and carers for people with dementia who had or had not spent time in hospital in the past month (RUD). A significant difference in ICECAP-O scores between carers of care receivers with high, medium and low levels of both the EQ-5D-5L tariff score and the EQ-5D-5L health problems index was observed. The ICECAP-O mean score was lower for carers of those with a lower QoL-AD or a higher CDR, and for carers of those with a lower DemQoL-U score.

### Multivariate analysis

The multivariate regression results are shown in Table 5. Due to missing data, only 389 observations were included in this analysis.

**Table 5:** Multivariate regression coefficients, confidence intervals and P values

	ICECAP-O tariff	ICECAP-O tariff (including countries)
**Carer**		
Age (years)	0.003 (− 0.005, 0.012)	0.002 (− 0.007, 0.010)
	0.47	0.73
Age^2^	− 0.00001 (− 0.0001, 0.0001)	− 0.000001 (− 0.00007, 0.00007)
	0.84	0.97
Gender (Female = 1 Male = 0)	− 0.025 (− 0.063, 0.012)	− 0.017 (− 0.055, 0.020)
	0.19	0.37
Education (years)	0.003 (− 0.001, 0.006)	0.001 (− 0.002, 0.005)
	0.13	0.42
Employed (employed = 1 unemployed = 0)	0.024 (− 0.019, 0.066)	0.018 (− 0.025, 0.061)
	0.28	0.42
PAI (range 6 to 30)	0.009 **(0.006, 0.012)**	0.009 **(0.006, 0.012)**
	< 0.01*	< 0.01*
**Care recipient**		
Age (years)	0.029 **(0.006, 0.052)**	0.031 **(0.008, 0.054)**
	0.02*	0.01*
Age^2^	− 0.0001 **(**− **0.0003,** − **0.00003)**	− 0.0002 **(**− **0.0004,** − **0.00004)**
	0.02*	0.01*
Gender (Female = 1 Male = 0)	0.010 (− 0.028, 0.048)	0.015 (− 0.024, 0.053)
	0.62	0.46
CDR (Range 0 to 5)	− 0.041 **(**− **0.075,** − **0.006)**	− 0.039 **(**− **0.073,** − **0.005)**
	0.02*	0.02*
EQ-5D-5L health problems index	− 0.005 **(**− **0.010,** − **0.0004)**	− 0.005 **(**− **0.010,** − **0.0003)**
	0.03*	0.04*
**Caregiving context**		
Spouse/partner (spouse or partner = 1 not spouse or partner = 0)	− 0.115 **(**− **0.174,** − **0.056)**	− 0.107 **(**− **0.165,** − **0.048)**
	< 0.01*	< 0.01*
Carer daily hours (hours*)*	0.002(− 0.0004, 0.005)	0.002 (− 0.0003, 0.0002)
	0.09	0.234
Unmet needs (CANE)	− 0.003 (− 0.010, 0.004)	− 0.004 (− 0.012, 0.004)
	0.42	0.32
DemQol-U	0.00821 (− 0.123, 0.139)	0.008 (− 0.124, 0.139)
	0.90	0.91
Perseverance time (range 0 to 6)	0.045 **(0.006, 0.012)**	0.047 **(0.016, 0.078)**
	0.01*	< 0.01*
Days in hospital	− 0.002 (− 0.011, 0.006)	− 0.002 (− 0.010, 0.007)
	0.61	0.68
Total home care services	− 0.0001 (− 0.0004, 0.0002)	− 0.00006 (− 0.0004, 0.0003)
	0.49	0.72
LSNS (range 0 to 36)	0.005 **(0.003, 0.009)**	0.005 **(0.003, 0.008)**
	< 0.01*	< 0.01*
Ireland		0.003 (− 0.069, 0.074)
		0.94
Italy		0.0004 (− 0.067, 0.068)
		0.99
The Netherlands		0.064 (− 0.003, 0.131)
		0.06
Norway		0.073 **(0.008, 0.139)**
		0.03*
Portugal		− 0.030 (− 0.097, 0.037)
		0.38
Sweden		0.043 (− 0.026, 0.112)
		0.22
United Kingdom		0.013 (− 0.051, 0.076)
		0.70
Constant	− 0.707 (− 0.123, 0.242)	− 0.722 (− 1.656, 0.212)
	0.14	0.13
*N*	389	389
*R*^2^	0.3359	0.3359

Germany is the reference country

Confidence intervals in bold indicate a significant effect

*P value of 0.05 or less

Several results can be derived from the multivariate regression. The age of the person with dementia, the relationship with the person with dementia, CDR, social isolation (LSNS) of the carer, the Positive Affect Index of the carer, and Perseverance Time all had a significant relationship with the ICECAP-O tariff score. Age of the person with dementia had a non-linear relationship with the carer ICECAP-O score, suggesting that when people with dementia reach roughly age 79 carer ICECAP-O scores stop increasing and start decreasing. Spouses or partners who care for the person with dementia had a significantly worse well-being than carers with other relationships with the recipient of care. A higher EQ-5D-5L health problems index had a significant negative relation with carer well-being. A higher CDR for the person with dementia score had a significant negative relation with carer well-being. A higher LSNS score had a significant positive relation with carer well-being, as did a higher PT score, while a higher PAI score had a significant positive relation with carer well-being. This can be summarized to mean that a lower severity of dementia and fewer health problems in the person with dementia, a better relationship with the person with dementia, and more perseverance time and less loneliness of the carer were associated with better well-being in the latter. The regression analysis in which countries were included shows that Norway has higher levels of carer well-being than the other countries in the sample.

## Discussion

The aim of this paper was to determine the validity of the ICECAP-O in a relatively large, eight-country population sample of informal carers for people living with dementia. Validation was performed using convergent and discriminant validity tests, followed by multivariate analysis. As hypothesized, there were significant moderate-to-strong correlations in the expected directions between the ICECAP-O scores and carers’ EQ-5D-5L utility tariff score and health problems index, EQ-VAS scores, as well as the CarerQol-7D and the CarerQol-VAS scores. The multivariate regressions showed that the age of the person with dementia, the EQ-5D-5L health index of the person with dementia, carer–patient relationship, care recipient CDR, carer LSNS Score, the carer PAI score, and Perseverance Time all had a significant relation with the carer ICECAP-O score. The fact that age of the person with dementia had a non-linear relationship with the carer’s ICECAP-O score may be explained by older people with dementia having more health and behavioural problems that were not captured in the multivariate regression. The reason for age of the person with the dementia being correlated with an increase in the ICECAP-O until age 79 is still a somewhat surprising result, perhaps explained by younger people wanting to take part in more activities or work than their older counterparts. Somewhat surprisingly, the ICECAP-O did not have a significant relationship with the number of daily care hours, even though it was assumed these would have an impact on carer well-being. This may be due to the selection of the sample as only people with mild to moderate dementia were included. The results also showed that living in certain countries may be of importance for the carer ICECAP-O scores.

Even though we presented the first validation of the ICECAP-O instrument in a sample of informal carers, our results were quite comparable to results from previous ICECAP-O validation studies [5, 12, 16, 19, 20]. While many addressed the specific dimensions of the ICECAP-O rather than the ICECAP-O tariff scores, several of the results found in our study were similar to those of previous studies. Almost all studies found that the ICECAP-O could discriminate effectively between groups of different ages [5, 12, 16, 20]. Additionally, all studies found moderate-to-strong convergent validity between the ICECAP-O and health (quite frequently using the EQ-5D as measure) although not necessarily for every dimension of both measures. Makai et al. [20] also found that the ICECAP-O could discriminate between older people who had more or fewer opportunities for social interaction, which is in line with the significance of the LSNS score in our analysis. Most previous validation studies conclude that the ICECAP-O may be a promising patient outcome measure in economic evaluations, although it may not completely cover physical health [18]. Based on the results of the above analysis, this paper comes to the same conclusion for the validity of the ICECAP-O in carers (of persons with dementia). An interesting finding is that in Sweden the ICECAP-O was not correlated with the EQ-5D-5L utility tariff score and health problems index. One reason for this may be that in our sample, the lowest EQ-5D-5L utility tariff score in Sweden is approximately 0.37, which is far higher than the lowest score from the full sample (− 0.1).

The main strength of this study is that it is the first to validate the ICECAP-O in carers: in a sample both large in size and country variety. While several validation studies of the ICECAP-O have been executed, they all used relatively small sample sizes and only focused on one country. The eight-country nature of this sample allowed a more comprehensive overview of the ICECAP-O’s validity in Europe (in carers). Another strength is that extensive data were provided on both carers and care receivers. None of the previous studies looked at convergent validity between the ICECAP-O and the CarerQol, CANE, RUD, PAI, QoL-AD and Perseverance Time. Moreover, this study was the first to validate the ICECAP-O in carers.

Some limitations of our study need to be mentioned as well. First is the lack of variation within the sample. Large percentages of both carers and care receivers were relatively healthy and most carers seemed to experience a relatively low care burden, which may be the result from selection bias as carers who experience a high care burden may be less likely to participate in the study. Therefore, a detailed analysis of validity of the ICECAP-O in those carers who are less healthy or feel higher care burden is not possible here. Second, carer proxy scores were used for some of the outcome measures for people with dementia (i.e. EQ-5D-5L, QoL-AD and DemQoL-U). The correlation of ICECAP-O scores with the harder to observe variables for persons with dementia (such as the DemQoL-U items) may be less reliable than those with more easily observed variables (such as the EQ-5D-5L items). In addition, due to the neurodegenerative nature of dementia and the stress experienced by carers, proxies may give more negative answers regarding care receivers’ health and well-being [42]. While this most likely does not affect our regression results, as from the measures of health of persons with dementia only CDR was used, it is worth bearing in mind for future studies. Another limitation is the use of UK value sets for both the ICECAP-O and EQ-5D-5L-related measures. This was done for consistency, as value sets were not available for all countries in the sample; however, it may be partially responsible for Sweden-specific EQ-5D-5L results being uncorrelated with the ICECAP-O. Finally, we used the ICECAP-O in the complete sample of carers. However, as can be derived from Table 2, nearly half of carers were younger than 65. The ICECAP-O (Older) was designed to capture the capability well-being of people age 65 and over. For people aged under 65, the ICECAP-A (Adults) [6]—which covers the five capability well-being dimensions attachment, stability, achievement, enjoyment and autonomy—would in principle be more suitable. This was not feasible in the current study. It is unclear how accurately the ICECAP-O measures the well-being of people aged under 65.

It has been shown in previous validation studies that the ICECAP-O is a worthy contender as a patient outcome measure in economic evaluations regarding care of the older people, due to its broad, well-being-focused nature. In this study, the ICECAP-O has shown good convergent and discriminant validity as a well-being outcome measure in carers of people with dementia. These findings suggest that the ICECAP-O potentially is a relevant and useful measure for economic evaluation in samples of elderly informal carers, especially when considering interventions that have impacts ‘beyond health’. If it is used in both carers and care receivers, this allows comparisons of outcomes across interventions and aggregation of outcomes within interventions. Before being able to recommend this, a number of important issues need to be resolved. First, the ICECAP-O needs to be further validated as an outcome measure among people with dementia and their carers. This would include linguistic validation of translations of the ICECAP-O, currently being analysed in Germany and Portugal as part of the Actifcare project [21]. It would be beneficial to conduct linguistic validations in other countries where psychometric validations have been conducted [48]. Second, future studies need to confirm our results and expand on them, to increase the evidence of the validity of the ICECAP-O. Third, a choice needs to be made whether the use of the generic ICECAP-O (which is aimed at older people) is to be preferred over the use of more carer-specific well-being measures, such as the CarerQol. While the results of the latter may be less easily aggregated with, for example, ICECAP outcomes in patients, they may provide more precise estimates of care-related quality of life and more detailed information. Finally, while the CarerQoL is aimed at carers (regardless of age), the ICECAP measures would need to be tailored to age groups of carers, which raises questions of aggregation and comparison of ICECAP-A and ICECAP-O scores.

Further research of the ICECAP-O in samples of informal carers for people with different chronic illnesses would also be useful. It would allow investigation into whether the ICECAP-O is also a valid measure and shows similar relationships to other outcomes in the context of diverse chronic illnesses. If the ICECAP-O is to be used as a well-being measure in economic evaluations, it would also be of interest to conduct further research into its sensitivity to change and Minimal Clinically Important Difference.

The ICECAP-O is a capability well-being measure that has been proven to be of use for economic evaluations of care of older people. This study adds that the ICECAP-O may be useful in economic evaluations of interventions considering elderly informal carers, where a broader measure of well-being is more relevant than a narrower health-related quality of life measure such as the EQ-5D-5L.

## Appendix 1

### Inclusion and exclusion criteria

#### Inclusion criteria

- The patient has a diagnosis of dementia meeting DSM IV TR criteria following an assessment by a clinical professional.
- The person with dementia has a Clinical Dementia Rating indicating mild or moderate degree of dementia (i.e. scores 1 or 2) or scores 24 or less on the MMSE.
- The patient is not receiving regular assistance from a paid worker with personal care, on account of his/her dementia, such as help with dressing/undressing; washing/bathing/showering; toileting; feeding/drinking; taking medication. (Note: ‘regular’ is defined as at least once per week; ‘paid worker’ includes those paid by health and social care services and those paid direct by the person and his/her family).
- A professional judges that additional assistance with personal care is likely to be considered/required within 1 year.
- The person with dementia has a carer who is able and willing to participate also and is in contact at least once per week. The carer does not have to be residing with the carer, they could be a relative, friend or neighbour in regular contact.

#### Exclusion criteria

- The person with dementia or their carer is not able to complete the assessments due to communication/language/hearing/understanding/literacy problems that cannot be compensated for.
- The person with dementia or their carer has a terminal condition or comorbidities (including long-standing severe mental illness) contributing to a significant level of disability.
- The person with dementia or their carer has a life-long learning disability or severe physical impairment that would prevent them from being able to complete the assessments.
- The person with dementia resides in a care home or nursing home or has been resident in a care home or nursing home (e.g. for respite) during the previous 6 months.
- The person with dementia has a diagnosis of alcohol-related dementia or of Huntington’s disease.

## Appendix 2

See Table 6.

**Table 6:** Summary statistics at baseline of continuous variables

	Mean	SD	Min	Max
**Carer**				
Age (years)	66.42	13.23	25	92
Education (years)	11.91	4.42	0	24
EQ-5D-5L utility tariff score	0.84	0.17	− 0.10	1
EQ-5D-5L health problems index	2.90	2.86	0	15
EQ-VAS	71.58	18.22	0	100
**Care receiver**				
Age (years)	77.77	7.83	47	98
Education (years)	9.85	4.49	0	25
EQ-5D-5L utility tariff score	0.71	0.21	− 0.11	1
EQ-5D-5L health problems index	5.43	3.46	0	16
EQ-VAS	61.13	19.77	0	100
Qol-AD sum score	31.36	6.00	16	50
DemQol-U tariff	0.82	0.11	0.46	0.99
**Caregiving context**				
Daily carer time (hours in a day)	6.33	5.96	0	23.23
Number of unmet needs (CANE)	1.78	2.04	0	12
Hospital days (RUD) last month	0.20	1.68	0	28
Practitioner visits (RUD) last month	1.57	2.12	0	17
Home service visits (RUD) last month	8.91	38.53	0	720
LSNS total score	16.60	5.55	2	30
CarerQol-7D utility tariff score	77.59	17.38	8.8	100
CarerQol-VAS	6.34	1.97	0	10

## Appendix 3

See Table 7.

**Table 7:** Country-specific spearman correlation

Instrument	ICECAP-O tariff	*P* value	ICECAP-O domains				
			Attachment	Security	Role	Enjoyment	Control
**Germany**							
EQ-5D- 5L utility tariff score	0.34	0.02	0.26	0.55	0.54	0.33	0.11
EQ-5D-5L health problems index	− 0.30	0.03	− 0.21	− 0.49	− 0.51	− 0.30	− 0.09
EQ-VAS	0.43	< 0.01	0.48	0.27	0.41	0.38	0.31
CarerQol tariff	0.62	< 0.01	0.56	0.61	0.48	0.55	0.47
CarerQol-VAS	0.65	< 0.01	0.70	0.46	0.51	0.51	0.50
**Ireland**							
EQ-5D-5L utility tariff score	0.64	< 0.01	0.35	0.59	0.48	0.46	0.54
EQ-5D-5L health problems index	− 0.68	< 0.01	− 0.36	− 0.59	− 0.44	− 0.45	− 0.58
EQ-VAS	0.39	< 0.01	0.20	0.32	0.038	0.28	0.30
CarerQol tariff	0.76	< 0.01	0.61	0.57	0.68	0.63	0.62
CarerQol-VAS	0.41	< 0.01	0.24	0.47	0.45	0.39	0.31
**Italy**							
EQ-5D-5L utility tariff score	0.56	< 0.01	0.34	0.37	0.39	0.56	0.48
EQ-5D-5L health problems index	− 0.56	< 0.01	− 0.40	− 0.36	− 0.45	− 0.54	− 0.47
EQ-VAS	0.53	< 0.01	0.45	0.26	0.33	0.49	0.36
CarerQol tariff	0.36	0.01	0.25	0.36	0.11	0.40	0.16
CarerQol-VAS	0.34	0.01	0.31	0.14	0.39	0.35	0.15
**The Netherlands**							
EQ-5D-5L utility tariff score	0.51	< 0.01	0.11	0.47	0.40	0.49	0.30
EQ-5D-5L health problems index	− 0.48	< 0.01	− 0.07	− 0.43	− 0.38	− 0.45	− 0.31
EQ-VAS	0.32	0.02	0.01	0.28	0.24	0.34	0.14
CarerQol tariff	0.45	< 0.01	0.27	0.28	0.43	0.52	0.25
CarerQol-VAS	0.51	< 0.01	0.32	0.34	0.46	0.51	0.16
**Norway**							
EQ-5D-5L utility tariff score	0.53	< 0.01	0.33	0.40	0.39	0.59	0.21
EQ-5D-5L health problems index	− 0.49	< 0.01	− 0.29	− 0.37	− 0.37	− 0.56	− 0.22
EQ-VAS	0.47	< 0.01	0.25	0.24	0.37	0.51	0.31
CarerQol tariff	0.47	< 0.01	0.40	0.22	0.33	0.48	-0.11
CarerQol-VAS	0.54	< 0.01	0.40	0.26	0.38	0.61	0.12
**Portugal**							
EQ-5D-5L utility tariff score	0.61	< 0.01	0.25	0.51	0.54	0.48	0.32
EQ-5D-5L health problems index	− 0.66	< 0.01	− 0.23	− 0.55	− 0.55	− 0.51	− 0.34
EQ-VAS	0.69	< 0.01	0.27	0.58	0.52	0.49	0.33
CarerQol tariff	0.52	< 0.01	0.43	0.41	0.54	0.39	0.12
CarerQol-VAS	0.63	< 0.01	0.45	0.38	0.64	0.57	0.23
**Sweden**							
EQ-5D-5L utility tariff score	0.21	0.14	0.05	0.31	0.10	0.12	0.14
EQ-5D-5L health problems index	− 0.17	0.25	− 0.04	− 0.29	− 0.05	− 0.09	− 0.12
EQ-VAS	0.39	< 0.01	0.19	0.26	0.09	0.37	0.13
CarerQol tariff	0.52	< 0.01	0.11	0.36	0.28	0.42	0.34
CarerQol-VAS	0.49	< 0.01	0.35	0.47	0.35	0.44	0.11
**United Kingdom**							
EQ-5D-5L utility tariff score	0.36	< 0.01	0.17	0.31	0.43	0.34	0.24
EQ-5D-5L health problems index	− 0.36	< 0.01	− 0.18	− 0.30	− 0.43	− 0.31	− 0.25
EQ-VAS	0.32	< 0.01	0.13	0.40	0.40	0.41	-0.06
CarerQol tariff	0.48	< 0.01	0.47	0.49	0.54	0.34	0.19
CarerQol-VAS	0.62	< 0.01	0.55	0.62	0.57	0.53	0.07

## References

[1] Patterson C (2018). World Alzheimer Report 2018—The state of the art of dementia research: New frontiers.

[2] Wimo A, Gauthier S, Prince M (2015). Global estimates of informal care.

[3] Bobinac A, Van Exel N, Job A, Rutten FF, Brouwer WB (2010). Caring for and caring about: disentangling the caregiver effect and the family effect. Journal of Health Economics.

[4] Vedhara K, Cox NK, Wilcock GK, Perks P, Hunt M, Anderson S (1999). Chronic stress in elderly carers of dementia patients and antibody response to influenza vaccination. The Lancet.

[5] Makai P, Beckebans F, van Exel J, Brouwer WB (2014). Quality of life of nursing home residents with dementia: validation of the German version of the ICECAP-O. PLoS ONE.

[6] Al-Janabi H, Flynn TN, Coast J (2012). Development of a self-report measure of capability wellbeing for adults: The ICECAP-A. Quality of Life Research.

[7] Bérenger V, Verdier-Chouchane A (2007). Multidimensional measures of well-being: Standard of living and quality of life across countries. World Development.

[8] Al-Janabi H, Coast J, Flynn TN (2008). What do people value when they provide unpaid care for an older person? A meta-ethnography with interview follow-up. Social Science and Medicine.

[9] Brouwer W, Van Exel N, Van Gorp B, Redekop W (2006). The CarerQol instrument: A new instrument to measure care-related quality of life of informal caregivers for use in economic evaluations. Quality of Life Research.

[10] Netten A, Burge P, Malley J, Potoglou D, Towers A, Brazier J (2012). Outcomes of social care for adults: Developing a preference-weighted measure. Health Technology Assessment.

[11] Grewal I, Lewis J, Flynn T, Brown J, Bond J, Coast J (2006). Developing attributes for a generic quality of life measure for older people: Preferences or capabilities?. Social Science and Medicine.

[12] Coast J, Peters TJ, Natarajan L, Sproston K, Flynn T (2008). An assessment of the construct validity of the descriptive system for the ICECAP capability measure for older people. Quality of Life Research.

[13] Makai P, Looman W, Adang E, Melis R, Stolk E, Fabbricotti I (2015). Cost-effectiveness of integrated care in frail elderly using the ICECAP-O and EQ-5D: Does choice of instrument matter?. The European Journal of Health Economics.

[14] Bowling A (2014). Research methods in health: Investigating health and health services.

[15] Coast J, Flynn TN, Natarajan L, Sproston K, Lewis J, Louviere JJ (2008). Valuing the ICECAP capability index for older people. Social Science and Medicine.

[16] Flynn TN, Chan P, Coast J, Peters TJ (2011). Assessing quality of life among British older people using the ICEPOP CAPability (ICECAP-O) measure. Applied Health Economics and Health Policy.

[17] Hackert MQ, van Exel J, Brouwer WB (2017). Valid outcome measures in Care for Older People: Comparing the ASCOT and the ICECAP-O. Value in Health.

[18] Hackert MQ, van Exel J, Brouwer WB (2018). Does the ICECAP-O cover the physical, mental and social functioning of older people in the UK?. Quality of Life Research.

[19] Makai P, Brouwer WB, Koopmanschap MA, Nieboer AP (2012). Capabilities and quality of life in Dutch psycho-geriatric nursing homes: an exploratory study using a proxy version of the ICECAP-O. Quality of Life Research.

[20] Makai P, Koopmanschap MA, Brouwer WB, Nieboer AA (2013). A validation of the ICECAP-O in a population of post-hospitalized older people in the Netherlands. Health and Quality of Life Outcomes.

[21] Kerpershoek L, De Vugt M, Wolfs C, Jelley H, Orrel M, Woods B (2016). Access to timely formal dementia care in Europe: Protocol of the Actifcare (ACcess to Timely Formal Care) study. BMC Health Services Research.

[22] O’Shea E, Hopper L, Marques M, Gonçalves-Pereira M, Woods B, Jelley H (2018). A comparison of self and proxy quality of life ratings for people with dementia and their carers: A European prospective cohort study. Aging and Mental Health.

[23] Handels RL, Sköldunger A, Bieber A, Edwards RT, Gonçalves-Pereira M, Hopper L (2018). Quality of life, care resource use, and costs of dementia in 8 European countries in a cross-sectional cohort of the Actifcare Study. Journal of Alzheimer’s Disease.

[24] Herdman M, Gudex C, Lloyd A, Janssen M, Kind P, Parkin D (2011). Development and preliminary testing of the new five-level version of EQ-5D (EQ-5D-5L). Quality of Life Research.

[25] Smith VK, Mansfield C, Strong A (2014). How should the health benefits of food safety programs be measured. Advances in Health Economics and Health Services Research.

[26] Kraijo H, Brouwer W, de Leeuw R, Schrijvers G, van Exel J (2014). The perseverance time of informal carers of dementia patients: Validation of a new measure to initiate transition of care at home to nursing home care. Journal of Alzheimer’s Disease.

[27] Lubben J, Blozik E, Gillmann G, Iliffe S, von Renteln Kruse W, Beck JC (2006). Performance of an abbreviated version of the Lubben Social Network Scale among three European community-dwelling older adult populations. The Gerontologist.

[28] Tan JE, Strauss E, Sherman EM (2011). Clinical dementia rating. encyclopedia of clinical neuropsychology.

[29] Mulhern B, Rowen D, Brazier J, Smith S, Romeo R, Tait R (2013). Development of DEMQOL-U and DEMQOL-PROXY-U: Generation of preference-based indices from DEMQOL and DEMQOL-PROXY for use in economic evaluation. Health Technology Assessment.

[30] Rowen D, Mulhern B, Banerjee S, van Hout B, Young TA, Knapp M (2012). Estimating preference-based single index measures for dementia using DEMQOL and DEMQOL-Proxy. Value in Health.

[31] Logsdon RG, Gibbons LE, McCurry SM, Teri L (1999). Quality of life in Alzheimer’s disease: Patient and caregiver reports. Journal of Mental Health and Aging.

[32] Wimo A, Jonsson L, Zbrozek A (2010). The resource utilization in dementia (RUD) instrument is valid for assessing informal care time in community-living patients with dementia. The Journal of Nutrition, Health and Aging.

[33] Reynolds T, Thornicroft G, Abas M, Woods B, Hoe J, Leese M (2000). Camberwell assessment of need for the elderly (CANE): Development, validity and reliability. The British Journal of Psychiatry.

[34] Cohen J (1988). Set correlation and contingency tables. Applied Psychological Measurement.

[35] Feng Y, Devlin N, Shah K, Mulhern B, Van Hout B (2016). New methods for modelling EQ-5D-5L value sets: An application to English data. Health Economics.

[36] Schneider U, Trukeschitz B, Mühlmann R, Ponocny I (2013). “Do I stay or do I go?”—Job change and labor market exit intentions of employees providing informal care to older adults. Health Economics.

[37] Salin S, Kaunonen M, Åstedt-Kurki P (2009). Informal carers of older family members: How they manage and what support they receive from respite care. Journal of Clinical Nursing.

[38] Lyonette C, Yardley L (2003). The influence on carer wellbeing of motivations to care for older people and the relationship with the care recipient. Ageing and Society.

[39] Freyne A, Kidd N, Coen R, Lawlor BA (1999). Burden in carers of dementia patients: Higher levels in carers of younger sufferers. International Journal of Geriatric Psychiatry.

[40] Markowitz JS, Gutterman EM, Sadik K, Papadopoulos G (2003). Health-related quality of life for caregivers of patients with Alzheimer disease. Alzheimer Disease and Associated Disorders.

[41] Greaves CJ, Farbus L (2006). Effects of creative and social activity on the health and well-being of socially isolated older people: Outcomes from a multi-method observational study. The Journal of the Royal Society for the Promotion of Health.

[42] Neumann PJ, Araki SS, Gutterman EM (2000). The use of proxy respondents in studies of older adults: Lessons, challenges, and opportunities. Journal of the American Geriatrics Society.

[43] Rabin R, Charro FD (2001). EQ-SD: A measure of health status from the EuroQol Group. Annals of Medicine.

[44] Xie F, Pullenayegum E, Gaebel K, Oppe M, Krabbe PF (2014). Eliciting preferences to the EQ-5D-5L health states: Discrete choice experiment or multiprofile case of best–worst scaling?. The European Journal of Health Economics.

[45] EuroQol—How to use EQ-5D. http://www.euroqol.org/about-eq-5d/how-to-use-eq-5d.html. Accessed 21 Dec 2016.

[46] Hoefman RJ, van Exel J, Rose JM, van de Wetering EJ, Brouwer WB (2014). A discrete choice experiment to obtain a tariff for valuing informal care situations measured with the CarerQol instrument. Medical Decision Making.

[47] Morris JC (1993). The clinical dementia rating (CDR): Current version and scoring rules. Neurology..

[48] Sarabia-Cobo CM, Parás-Bravo P, Amo-Setién FJ, Alconero-Camarero AR, Sáenz-Jalón M, Torres-Manrique B, Paz-Zulueta M (2017). Validation of the Spanish version of the ICECAP-O for nursing home residents with dementia. PLoS ONE.

